# Association of healthy sleep pattern with lower risk of acute myocardial infarction mortality among people with diabetes: A prospective cohort study

**DOI:** 10.1111/1753-0407.13528

**Published:** 2024-04-10

**Authors:** Min Du, Min Liu, Jue Liu

**Affiliations:** ^1^ Department of Epidemiology and Biostatistics, School of Public Health Peking University Beijing China; ^2^ Key Laboratory of Epidemiology of Major Diseases (Peking University) Ministry of Education Beijing China; ^3^ PKU Institute for Global Health and Development Peking University Beijing China

## Abstract

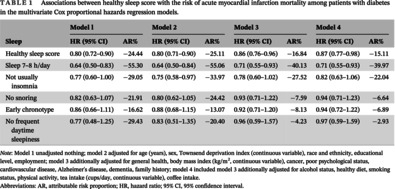

## Graphical abstract

Highlights

- This large cohort study investigated a potential association between sleep pattern and acute myocardial infarction mortality among individuals with diabetes.
- Healthy sleep score showed a linear relationship with acute myocardial infarction mortality among people with diabetes.
- Of five sleep habits, adequate sleep duration was associated with lowest risk of acute myocardial infarction mortality.

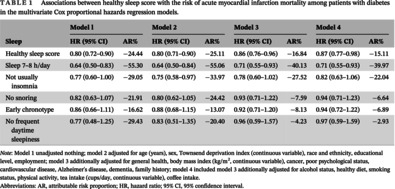

To the Editor,

Diabetes mellitus (DM) has substantial and sustained effects on postacute myocardial infarction (AMI) all‐cause mortality at short‐term, midterm and long‐term follow‐up.^1^ Meanwhile, sleep was considered as a key influencing factor of death in recent years. One cohort study showed that healthy sleep was associated with lower risk of cardiovascular disease mortality among general population and people with DM.^2^, ^3^ However, the evidence on protective factors for AMI mortality among DM patients was limited, especially for sleep pattern. In addition, previous studies did not control important covariates that affect the progression of AMI, DM, and sleep simultaneously including smoking and physical activity.^4^ To address these gaps, we investigated the association of sleep pattern with risk of mortality from AMI among patients with DM by analyzing the well‐characterized UK Biobank cohort study.

## 1 METHODS

We obtained data from a large prospective cohort study—The UK Biobank (application 79 114), recruiting over 500 000 participants aged 37–73 years between 2006 and 2021.^5^, ^6^ The UK Biobank study has ethical approval derived from Northwest Multi‐Center Research Ethics Committee (reference no. 21/NW/0157). All participants provided written informed consent to participate. The final study included 20 177 participants, after excluding individuals without DM (*n* = 475 480) and those with missing variables for sleep pattern (*n* = 5364) and other covariates (*n* = 1393).

All self‐reported sleep behaviors including sleep duration, chronotype, insomnia symptoms, snoring, and daytime sleepiness were obtained by using a touchscreen questionnaire at baseline visit in the UK Biobank study. Sleep 7–8 h/day, not usually insomnia, no snoring, early chronotype, and no frequent daytime sleepiness all were low‐risk sleep behaviors.^2^, ^3^ The scores of all five components were summed to create a healthy sleep score that ranged from 0 to 5. A higher healthy sleep score indicated a healthier sleep pattern. Mortality data were obtained from death certificates according to the National Health Service Information Centre (England and Wales) and the National Health Service Central Register Scotland (Scotland).

We used Cox proportional hazards regression models to estimate the hazard ratios (HRs) and 95% confidence intervals (95% CI). In addition to giving the HR (95% CI) in an unadjusted model 1, we also adjusted covariates step by step in model 2, which adjusted demographic characteristics; model 3, which additionally adjusted health status; and model 4, which further adjusted lifestyle habits. Dose–response relationships were examined using restricted cubic spline analysis with three knots. Additionally, we calculated the attributable risk proportion. Two‐sided *p* values <.05 were considered statistically significant.

## 2 RESULTS

**FIGURE 1:**
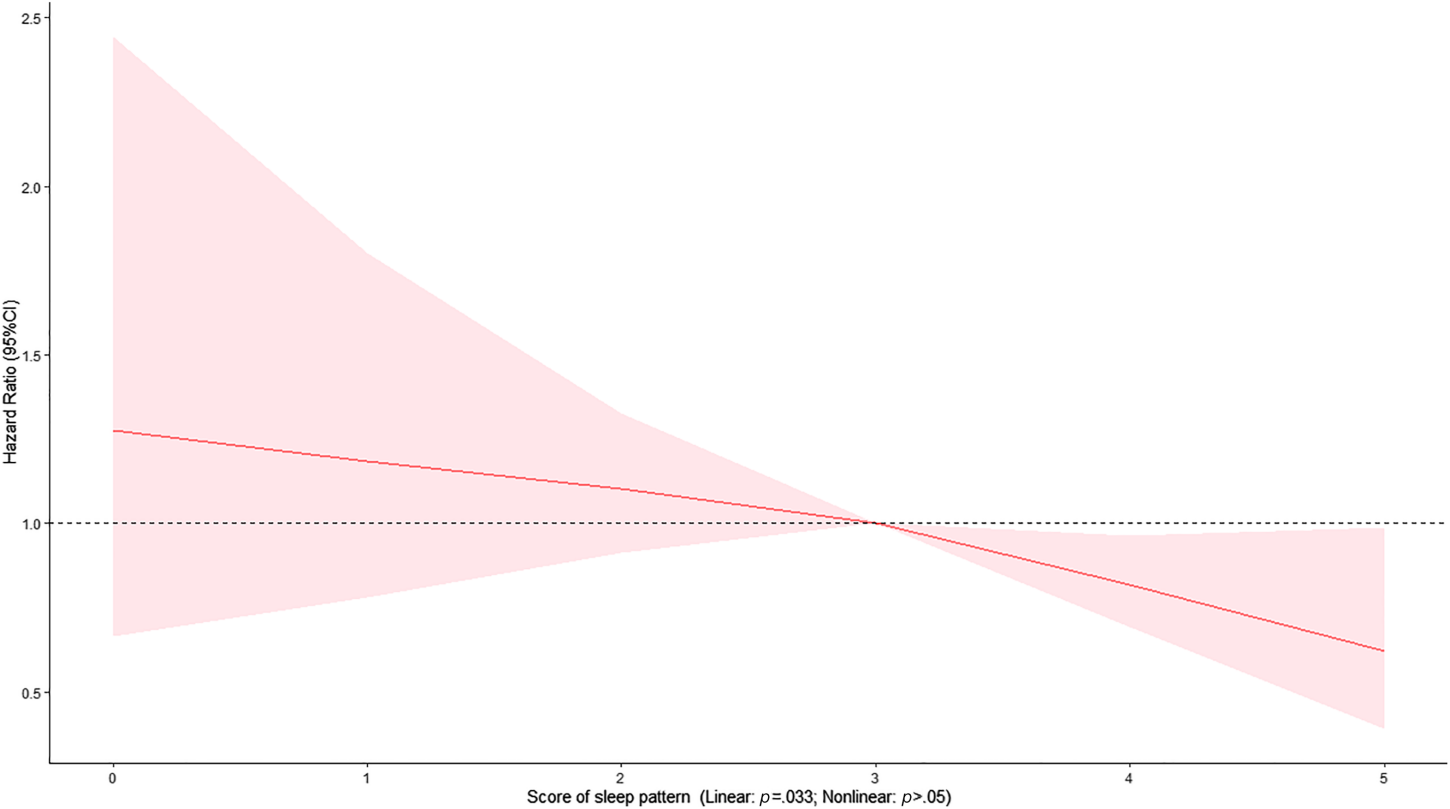
Multivariable Cox regression model with penalized splines on association of healthy sleep scores and acute myocardial infarction mortality among diabetes patients. Model adjusted for age (years), sex, Townsend deprivation index (continuous variable), race and ethnicity, educational level, employment, general health, BMI (kg/m^2^, continuous variable), cancer, poor psychological status, cardiovascular disease, Alzheimer's disease, dementia, family history, alcohol status, healthy diet, smoking status, physical activity, tea intake (cups/day, continuous variable), coffee intake. BMI, body mass index; HR, hazard ratio; 95% CI, 95% confidence interval. The red line represents hazard ratio; the pink shadow represents 95% confidence ratio.

At the baseline, the median age of the 20 177 participants with DM was 61.00 (interquartile range [IQR]: 55.00, 65.00) years, and 60.2% of participants were female. During a median follow‐up of 12.30 years (IQR: 11.45–13.18 years), 235 participants died of AMI. One point increase of the healthy sleep score was associated with 2%–23% lower risks of AMI mortality (HR 0.87; 95% CI: 0.77–0.98) (Table 1). Cox models with penalized splines showed statistically significant linear associations of sleep scores with AMI mortality (*p* = .033) (Figure 1). In addition, adequate sleep duration was associated with lower risk of AMI mortality (HR 0.71; 95% CI: 0.55–0.93), after adjustment for covariates with 39.97% reduced attributable risk proportion (Table 1).

**TABLE 1:** Associations between healthy sleep score with the risk of acute myocardial infarction mortality among patients with diabetes in the multivariate Cox proportional hazards regression models.

Sleep	Model l		Model 2		Model 3		Model 4	
	HR (95% CI)	AR%	HR (95% CI)	AR%	HR (95% CI)	AR%	HR (95% CI)	AR%
Healthy sleep score	0.80 (0.72–0.90)	−24.44	0.80 (0.71–0.90)	−25.11	0.86 (0.76–0.96)	−16.84	0.87 (0.77–0.98)	−15.11
Sleep 7–8 h/day	0.64 (0.50–0.83)	−55.30	0.64 (0.50–0.84)	−55.06	0.71 (0.55–0.93)	−40.13	0.71 (0.55–0.93)	−39.97
Not usually insomnia	0.77 (0.60–1.00)	−29.05	0.75 (0.58–0.97)	−33.97	0.78 (0.60–1.02)	−27.52	0.82 (0.63–1.06)	−22.04
No snoring	0.82 (0.63–1.07)	−21.91	0.80 (0.62–1.05)	−24.42	0.93 (0.71–1.22)	−7.59	0.94 (0.71–1.23)	−6.64
Early chronotype	0.86 (0.66–1.11)	−16.62	0.88 (0.68–1.15)	−13.07	0.92 (0.71–1.20)	−8.13	0.94 (0.72–1.22)	−6.89
No frequent daytime sleepiness	0.77 (0.48–1.25)	−29.43	0.83 (0.51–1.35)	−20.40	0.96 (0.59–1.57)	−4.23	0.97 (0.59–1.59)	−2.93

*Note*: Model 1 unadjusted nothing; model 2 adjusted for age (years), sex, Townsend deprivation index (continuous variable), race and ethnicity, educational level, employment; model 3 additionally adjusted for general health, body mass index (kg/m^2^, continuous variable), cancer, poor psychological status, cardiovascular disease, Alzheimer's disease, dementia, family history; model 4 included model 3 additionally adjusted for alcohol status, healthy diet, smoking status, physical activity, tea intake (cups/day, continuous variable), coffee intake.

Abbreviations: AR, attributable risk proportion; HR, hazard ratio; 95% CI, 95% confidence interval.

## 3 COMMENT

To our knowledge, this is the first large prospective study investigating the association of healthy sleep pattern with AMI mortality among DM patients. In our study of 20 177 participants from UK Biobank, a higher healthy sleep score was associated with lower risk of AMI mortality. Of five sleep habits, adequate sleep duration was associated with lowest risk of AMI mortality among diabetes patients. Several studies reported that some sleep habits were associated with the survival of AMI patients.^3^, ^7^ Generally, sleep behaviors are multidimensional and interact with each other. Insufficient nighttime sleep duration or insomnia may highly correlate with daytime sleepiness and drowsiness; thus, a holistic analysis of multiple sleep behaviors was essential. In our study, we used healthy sleep scores, not the single sleep behavior, to make interpretation of results easier and form a sound sleep guidance program in clinical and public health practice. Previous studies found that sleep disturbance was associated with higher levels of C‐reactive protein (CRP) and interleukin‐6, whereas shorter and longer sleep duration both were associated with higher levels of CRP.^8^ Furthermore, inflammation worsened the progression of diabetes, and dysglycemia caused significant endothelial dysfunction and upregulation of inflammation within the vasculature and finally increased the risk of AMI morality.^9^, ^10^

Our study has several major strengths. Firstly, it was the first cohort study with large sample size to report the association between multifaceted sleep habits and AMI mortality among DM patients. Second, we used death records from register data to make them accurate. Although self‐reported sleep habits were important and more convenient for further developing targeted interventions and monitoring, it still brought recall bias. Besides, other important confounders, such as inflammation could not be controlled in our study. The objective measure of sleep and adjustment of important confounders were needed to confirmed our findings in the future. In addition, the association may be different between two main categories of DM (type 1 and type 2). Future studies should explore the possible difference based on categories of DM.

## AUTHOR CONTRIBUTIONS

Min Du and Jue Liu designed the study, involved in data curation. Min Du did formal analysis, drafted the manuscript. Min Du, Jue Liu, and MinLiu revised manuscript critically for important intellectual content. All authors contributed to manuscript revision and read and approved the final version.

## FUNDING INFORMATION

This work was supported by in part by the National Natural Science Foundation of China (72122001, 72211540398).

## DISCLOSURE

The authors have declared that no competing interests exist. The funders had no role in study design, data collection and analysis, decision to publish, or preparation of the manuscript.

## CONSENT FOR PUBLICATION

Not applicable.

## Data Availability

The UK Biobank datasets are openly available by submitting a data request proposal from https://www.ukbiobank.ac.uk/ (accessed on 9 June 2022).
